# Supplementary material for: Patterns of symptoms possibly indicative of cancer and associated help-seeking behaviour in a large sample of United Kingdom residents—The USEFUL study
Source: PLoS One. 2020 Jan 24;15(1):e0228033. doi: 10.1371/journal.pone.0228033 (PMC6980617; doi:10.1371/journal.pone.0228033)
Supplement: S1 Table — (DOCX) [file pone.0228033.s001.docx]

**S1 Table. Response rates in Scotland and England**

| **Scotland** | | | | | | | |
| --- | --- | --- | --- | --- | --- | --- | --- |
| **Demographic group** | **Total n** | **Sub-group** | **Responder** | | **Non-responder** | | **Chi square†** |
|  |  |  | **n** | **%** | **n** | **%** |  |
| **Sex** | 25000 | Male | 4056 | 33.5 | 8055 | 66.5 | 40.3, df=1,  p<0.001 |
|  |  | Female | 4812 | 37.3 | 8077 | 62.7 |  |
| **Age group** | 25000 | 50-54 | 1646 | 30.4 | 3777 | 69.6 | 3.1, df=1,  p=0.08 |
|  |  | 55-59 | 1650 | 33.9 | 3224 | 66.1 |  |
|  |  | 60-64 | 1686 | 40.2 | 2506 | 59.8 |  |
|  |  | 65-69 | 1673 | 42.4 | 2272 | 57.6 |  |
|  |  | 70-74 | 1110 | 40.0 | 1664 | 60.0 |  |
|  |  | 75-79 | 658 | 32.7 | 1355 | 67.3 |  |
|  |  | 80 | 445 | 25.0 | 1334 | 75.0 |  |
| **Urban Rural Classification Scotland** | 24940 | Large urban areas | 3234 | 34.0 | 6284 | 66.0 | 31.8, df=1,  p<0.001 |
|  |  | Other urban areas | 1434 | 37.2 | 2426 | 62.8 |  |
|  |  | Accessible small towns | 640 | 30.2 | 1482 | 69.8 |  |
|  |  | Remote small towns | 1569 | 35.4 | 2860 | 64.6 |  |
|  |  | Accessible rural | 959 | 37.4 | 1604 | 62.6 |  |
|  |  | Remote rural | 1013 | 41.4 | 1435 | 58.6 |  |
| **Deprivation Decile Scotland** | 24940 | 1 (most deprived) | 223 | 16.7 | 1111 | 83.3 | 441.4, df=1, p<0.001 |
|  |  | 2 | 301 | 25.2 | 895 | 74.8 |  |
|  |  | 3 | 465 | 27.6 | 1220 | 72.4 |  |
|  |  | 4 | 268 | 26.9 | 727 | 73.1 |  |
|  |  | 5 | 681 | 32.3 | 1425 | 67.7 |  |
|  |  | 6 | 1175 | 36.3 | 2058 | 63.7 |  |
|  |  | 7 | 1100 | 37.8 | 1813 | 62.2 |  |
|  |  | 8 | 1263 | 38.8 | 1989 | 61.2 |  |
|  |  | 9 | 1348 | 40.4 | 1990 | 59.6 |  |
|  |  | 10 (least deprived) | 2025 | 41.4 | 2863 | 58.6 |  |
| **England** | | | | | | | |
| **Sex** | 25000 | Male | 3689 | 30.3 | 8499 | 69.7 | 20.7, df=1, p<0.001 |
|  |  | Female | 4221 | 32.9 | 8591 | 67.1 |  |
| **Age group** | 25000 | 50-54 | 1112 | 24.6 | 3409 | 75.4 | 44.4, df=1, p<0.001 |
|  |  | 55-59 | 1156 | 28.0 | 2967 | 72.0 |  |
|  |  | 60-64 | 1383 | 35.8 | 2475 | 64.2 |  |
|  |  | 65-69 | 1608 | 36.8 | 2756 | 63.2 |  |
|  |  | 70-74 | 1212 | 37.3 | 2036 | 62.7 |  |
|  |  | 75-79 | 732 | 32.1 | 1550 | 67.9 |  |
|  |  | 80 | 707 | 27.2 | 1897 | 72.8 |  |
| **Urban Rural Classification England** | 24878 | Urban with city and town | 2367 | 25.0 | 7108 | 75.0 | 294.4, df=1, p<0.001 |
|  |  | Urban with significant rural | 3201 | 34.5 | 6072 | 65.5 |  |
|  |  | Largely rural | 2293 | 37.4 | 3837 | 62.6 |  |
| **Deprivation Decile England** | 24878 | 1 (most deprived) | 136 | 15.6 | 735 | 84.4 | 230, df=1, p<0.001 |
|  |  | 2 | 373 | 24.0 | 1184 | 76.0 |  |
|  |  | 3 | 293 | 24.3 | 914 | 75.7 |  |
|  |  | 4 | 436 | 26.2 | 1226 | 73.8 |  |
|  |  | 5 | 918 | 30.7 | 2077 | 69.3 |  |
|  |  | 6 | 654 | 31.4 | 1431 | 68.6 |  |
|  |  | 7 | 1305 | 34.4 | 2489 | 65.6 |  |
|  |  | 8 | 1382 | 34.9 | 2577 | 65.1 |  |
|  |  | 9 | 1335 | 34.6 | 2528 | 65.4 |  |
|  |  | 10 (least deprived) | 1029 | 35.7 | 1856 | 64.3 |  |
