# Supplementary material for: Patterns of symptoms possibly indicative of cancer and associated help-seeking behaviour in a large sample of United Kingdom residents—The USEFUL study
Source: PLoS One. 2020 Jan 24;15(1):e0228033. doi: 10.1371/journal.pone.0228033 (PMC6980617; doi:10.1371/journal.pone.0228033)
Supplement: S2 Table — (DOCX) [file pone.0228033.s002.docx]

| **S2 Table. Proportion and chance of participants in different subgroups having experienced at least one symptom possibly indicative of different cancers in the last year** | | | | | | | | | | |
| --- | --- | --- | --- | --- | --- | --- | --- | --- | --- | --- |
|  | **At least one symptom of possible:** | | | | | | | | | |
|  |  | | | | | | | | | |
|  | **Upper GIT cancer** | | **Lung cancer** | | **Colorectal cancer** | | **Breast cancer** | | **Non-specific cancer** | |
| **Sub-group†** | **% (99% CI)** | **AOR‡**  **(99% CI)** | **% (99% CI)** | **AOR‡**  **(99% CI)** | **% (99% CI)** | **AOR‡**  **(99% CI)** | **% (99% CI)** | **AOR‡**  **(99% CI)** | **% (99% CI)** | **AOR‡**  **(99% CI)** |
| **Sex** (Male)^R^ | 25.8 (24.5-27.1) |  | 35.8 (34.4-37.2)  310 334 (31 |  | 16.1 (15.0-17.2) |  | 0.4 (0.2-0.6) |  | 17.6 (16.5-18.7) |  |
| Female | 32.5 (31.2-33.8) | **1.37 (1.23-1.52)** | 34.4 (33.1-35.7) | 0.92 (0.83-1.01) | 18.2 (17.2-19.3) | **1.15 (1.01-1.31)** | 3.6 (3.1-4.1) | **14.5 (7.52-27.93)** | 24.5 (23.3-25.7) | **1.59 (1.40-1.79)** |
| **Age group** (50-59)^R^ | 32.4 (30.8-34.0) |  | 32.0 (30.4-33.6) | 0.99 (0.86-1.14) | 17.8 (16.5-19.1)  11.9 ( ()  1007  606  296 |  | 2.8 (2.3-3.4)  66  20 | 0.66 | 25.1 (23.6-26.6) |  |
| 60-69 | 28.5 (27.0-30.0) | **0.77 (0.69-0.89)** | 33.7 (32.2-35.2) | 0.99 (0.86-1.14) | 15.9 (14.7-17.1) | **0.78 (0.65-0.92)** | 1.8 (1.4-2.2) | 0.66 (0.41-1.04) | 18.5 (17.2-19.8) | **0.67 (0.57-0.78)** |
| 70-79 | 27.1 (25.2-29.0) | **0.70 (0.58-0.84)** | 38.0 (37.0-40.1) | 1.12 (0.94-1.35) | 16.3 (14.7-17.9) | **0.74 (0.59-0.93)** | 1.8 (1.2-2.4) | 0.70 (0.38-1.29) | 18.5 (16.7-20.1) | **0.66 (0.53-0.82)** |
| 80+ | 27.2 (23.8-30.6) | **0.67 (0.51-0.88)** | 47.4 (43.6-51.2) | 1.49 (1.16-1.91) | 25.7 (22.4-29.0) | 1.26 (0.94-1.70) | 1.7 (0.7-2.7) | 0.56 (0.21-1.49) | 27.6 (24.2-301.0) | 1.02 (0.77-1.36) |
| **Marital status** (Single)^R^ | 28.6 (25.1-32.1) |  | 38.5 (34.7-42.3)  1315 |  | 17.0 (14.1-19.9) |  | 1.7 (0.7-2.7) |  | 24.5 (21.2-27.8) |  |
| Married/living together | 28.5 (27.5-29.6) | 1.12 (0.97-1.47) | 32.8 (31.7-33.9) | 0.86 (0.71-1.04) | 16.4 (15.5-17.3) | 1.05 (0.82-1.34) | 2.0 (1.7-2.3) | 1.09 (0.54-2.19) | 19.5 (18.6-20.4) | 0.95 (0.76-1.19) |
| No longer married | 33.4 (31.2-35.6) | 1.26 (1.01-1.58) | 42.4 (40.1-44.7) | 1.04 (0.84-1.29) | 20.6 (18.7-22.5) | 1.10 (0.85-1.43) | 2.7 (2.0-3.5) | 1.23 (0.59-2.55) | 27.3 (25.2-29.4) | 1.12 (0.88-1.43) |
| **Social support** (Low)^R^  Medium  High | 33.8 (31.2-35.6)  30.1 (28.5-31.7)  28.9 (27.7-30.1) | 0.85 (0.68-1.06)  0.81 (0.65-1.00) | 43.1 (38.9-47.3)  35.1 (33.5-36.8)  34.3 (33.0-35.6) | **0.77 (0.62-0.95)**  **0.78 (0.64-0.96)** | 20.2 (16.8-23.6)  18.0 (16.7-19.3)  16.5 (15.5-17.5) | 0.91 (0.70-1.17)  0.85 (0.66-1.10) | 0.9 (0.1-1.7)  2.1 (1.6-2.6)  2.3 (1.9-2.7) | 1.94 (0.70-5.39)  1.53 (0.56-4.21) | 32.5 (28.5-36.5)  22.0 (20.6-23.4)  19.8 (18.7-20.9) | **0.68 (0.54-0.86)**  **0.60 (0.47-0.75)** |
| **Education** (No qualifications)^R^ | 31.6 (29.0-34.2) |  | 39.8 (37.0-42.6) |  | 19.1 (16.9-21.3) |  | 1.5 (0.8-2.2) |  | 25.6 (23.1-28.1) | 0.93 (0.77-1.12) |
| Secondary school or equivalent | 30.6 (29.0-32.2) | 1.03 (0.87-1.23) | 36.1 (34.4-37.8) | 1.01 (0.85-1.19) | 17.2 (15.9-18.5) | 1.01 (0.82-1.25) | 2.1 (1.6-2.6) | 1.09 (0.58-2.03) | 22.6 (21.1-24.1) | 0.93 (0.77-1.12) |
| College/vocational courses and other | 32.1 (27.4-36.8) | 1.06 (0.80-1.42) | 38.3 (33.4-43.2) | 0.99 (0.75-1.31) | 17.4 (13.6-21.2) | 1.00 (0.70-1.41) | 2.9 (1.2-4.6) | 1.08 (0.41-2.86) | 23.3 (19.1-27.5) | 0.90 (0.65-1.23) |
| Professional qualification | 29.1 (27.3-30.9) | 0.99 (0.83-1.20) | 34.8 (32.9-36.7) | 1.00 (0.84-1.19) | 17.0 (15.5-18.5) | 1.09 (0.87-1.36) | 2.1 (1.5-2.7) | 1.10 (0.57-2.13) | 20.1 (18.5-21.7) | 0.93 (0.76-1.14) |
| Degree or postgraduate qualification | 26.9 (25.1-28.7) | 0.94 (0.77-1.14) | 31.0 (29.1-32.9) | 0.91 (0.75-1.09) | 16.3 (14.8-17.8) | 1.05 (0.83-1.32) | 2.6 (2.0-3.3) | 1.38 (0.71-2.69) | 18.3 (16.7-19.9) | 0.88 (0.71-1.10) |
| **Employment** (Working full-time)^R^ | 29.6 (27.7-31.5) |  | 31.3 (39.4-33.2) | 0.90 | 15.8 (14.3-17.3) |  | 1.9 (1.3-2.5) | 1. | 21.6 (19.9-23.3) |  |
| Working part-time | 30.2 (27.2-33.2) | 0.92 (0.76-1.11)  0. | 29.8 (26.9-32.8) | 0.90 (0.75-1.09) | 15.6 (13.3-17.9) | 0.97 (0.77-1.22) | 3.1 (2.0-4.2) | 1.12 (0.65-1.91) | 21.4 (18.8-24.0) | 0.86 (0.70-1.07) |
| Self-employed | 27.3 (24.0-30.6) | 0.97 (0.79-1.20) | 33.8 (30.3-37.3) | 1.03 (0.84-1.25) | 16.2 (13.5-18.9) | 1.06 (0.83-1.37) | 2.4 (1.3-3.5) | 1.48 (0.78-2.81) | 20.6 (17.6-23.6) | 1.01 (0.80-1.27) |
| Retired | 27.7 (26.5-28.9) | 0.89 (0.76-1.06) | 36.2 (34.9-37.5) | 0.92 (0.78-1.09) | 17.1 (16.1-18.1) | 1.02 (0.83-1.25) | 1.9 (1.5-2.3) | 1.12 (0.64-1.95) | 18.9 (17.8-20.0) | **0.73 (0.60-0.89**) |
| Unable to work due to illness/disability | 61.7 (55.6-67.8) | **2.52 (1.84-3.44)** | 67.8 (62.0-73.7) | **2.79 (2.02-3.85)** | 40.7 (34.6-46.9) | **2.85 (2.06-3.94)** | 3.8 (1.4-6.2) | 1.73 (0.70-4.28) | 62.4 (56.3-68.5) | **3.63 (2.62-5.03)** |
| Others not in paid employment | 33.5 (28.3-38.7) | 0.99 (0.75-1.33) | 35.0 (29.8-40.2) | 1.06 (0.80-1.42) | 18.5 (14.2-22.8) | 1.08 (0.76-1.52) | 3.3 (1.3-5.3) | 1.24 (0.56-2.71) | 26.1 (21.3-30.9) | 0.95 (0.69-1.30) |
| **Household Income** (< £15,000)^R^  £15,000-29,999  £30,000-49,999  >£50,000 | 34.4 (32.4-36.5)  28.1 (26.4-29.8)  28.4 (26.5-30.3)  26.4 (24.4-28.4) | **0.84 (0.73-0.97)**  0.87 (0.74-1.03)  **0.74 (0.61-0.90)** | 42.5 (40.4-44.6)  34.4 (32.6-36.2)  32.4 (30.4-34.4)  29.0 (26.9-31.1) | 0.85 (0.74-0.97)  0.87 (0.74-1.02)  **0.79 (0.66-0.94)** | 20.6 (18.9-22.4)  16.8 (15.4-18.2)  15.7 (14.2-17.3)  15.0 (13.4-16.7) | 0.90 (0.76-1.07)  0.83 (0.68-1.02)  **0.78 (0.62-0.97)** | 1.9 (1.3-2.5)  2.1 (1.6-2.7)  1.9 (1.3-2.5)  2.3 (1.6-3.0) | 1.47 (0.91-2.36)  1.28 (0.74-2.23)  1.52 (0.83-2.77) | 28.3 (26.4-30.2)  20.7 (19.2-22.3)  18.1 (16.5-19.7)  17.1 (15.4-18.8) | **0.84 (0.71-0.98)**  **0.73 (0.60-0.87)**  **0.63 (0.51-0.79**) |
| **Smoking status** (Never smoked)^R^  Ex-smoker  Current smoker | 27.8 (26.6-29.0)  31.0 (29.5-32.5)  32.9 (29.7-36.1) | 1.14 (1.02-1.27)  1.09 (0.91-1.30) | 31.3 (30.0-32.6)  38.5 (36.9-40.1)  42.6 (39.3-45.9) | **1.20 (1.08-1.33)**  **1.44 (1.21-1.70)** | 15.8 (14.8-16.8)  19.0 (17.7-20.3)  18.6 (16.0-21.2) | **1.19 (1.04-1.35)**  1.09 (0.88-1.35) | 2.3 (1.9-2.7)  2.0 (1.5-2.5)  1.6 (0.8-2.4) | 1.09 (0.78-1.52)  0.70 (0.36-1.38) | 19.2 (18.1-20.3)  22.2 (20.8-23.6)  29.6 (26.5-32.7) | **1.20 (1.06-1.36)**  **1.49 (1.23-1.81)** |
| **Rural Urban** (Scotland large urban)^R^  Scotland other urban areas  Scotland accessible small towns  Scotland remote small towns  Scotland accessible rural  Scotland remote rural  England urban with city and town  England urban with significant rural  England largely rural | 31.0 (28.9-33.1)  30.2 (27.1-33.3)  30.3 (25.6-35.0)  29.7 (26.7-32.7)  29.0 (25.2-32.8)  27.3 (23.7-30.9)  32.5 (30.0-35.0)  27.6 (25.6-29.6)  26.3 (23.9-28.7) | 0.87 (0.71-1.06  0.86 (0.65-1.13)  0.95 (0.78-1.15)  0.87 (0.70-1.10)  **0.77 (0.61-0.97)**  0.97 (0.81-1.16)  0.85 (0.72-1.00)  **0.77 (0.65-0.92)** | 37.1 (34.9-39.3)  34.7 (31.5-37.9)  35.0 (30.1-39.9)  34.7 (31.6-37.8)  33.2 (29.3-37.1)  33.0 (29.2-36.8)  38.9 (36.3-41.5)  32.4 (30.3-34.5)  34.1 (31.6-36.7) | 0.94 (0.77-1.14)  0.87 (0.66-1.13)  0.91(0.75-1.10)  0.92 (0.73-1.16)  0.90 (0.72-1.12)  0.98 (0.82-1.16)  **0.82 (0.70-0.96)**  0.87 (0.42-1.03) | 18.5 (16.7-20.3)  17.9 (15.3-20.5)  18.3 (14.4-22.2)  15.7 (13.3-18.1)  16.0 (13.0-19.1)  16.5 (13.5-19.5)  18.0 (16.0-20.0)  16.6 (14.9-18.3)  17.1 (15.1-19.1) | 0.96 (0.75-1.22)  0.93 (0.68-1.29)  0.78 (0.61-1.00)  0.88 (0.66-1.17)  0.89 (0.67-1.18)  0.91 (0.73-1.12)  0.92 (0.75-1.11)  0.91 (0.74-1.12) | 3.9 (3.0-4.8)  1.1 (0.4-1.8)  2.2 (0.7-3.7)  1.7 (0.7-2.5)  2.4 (1.1-3.7)  2.0 (0.9-3.1)  2.5 (1.7-3.3)  2.2 (1.5-2.9)  2.2 (1.4-3.0) | 0.55 (0.25-1.23)  1.04 (0.44-2.48)  0.98 (0.51-1.87)  1.20 (0.59-2.41)  0.70 (0.30-1.61)  1.33 (0.77-2.32)  1.18 (0.71-1.93)  1.18 (0.68-2.03) | 21.5 (19.6-23.4)  21.9 (19.1-24.7)  20.8 (16.7-25.0)  18.7 (16.2-21.2)  18.7 (15.5-21.9)  21.0 (17.7-24.3)  26.5 (24.1-28.8)  19.2 (17.4-21.0)  21.6 (19.4-23.8) | 1.07(0.85-1.35)  0.85 (0.62-1.17)  0.90 (0.71-1.13)  0.93 (0.71-1.23)  1.10 (0.85-1.42)  1.23 (1.01-1.51)  1.02 (0.85-1.23)  1.06 (0.87-1.30) |
| **Diagnosis of specified condition** (No)^R^  Yes | 9.2 (8.0-10.5)  32.5 (31.5-33.6) | **2.33 (2.03-2.68)** | 19.2 (17.5-20.9)  39.3 (38.2-40.4) | **2.53 (2.21-2.89)** | 10.4 (9.1-11.7)  19.1 (18.2-20.0) | **1.87 (1.58-2.21)** | 1.5 (1.0-2.0)  2.3 (2.0-2.6) | **1.55 (1.01-2.38)** | 10.6 (9.3-11.9)  24.2 (23.2-25.2) | **2.64 (2.23-3.12)** |

CI= confidence interval, AOR= adjusted odds ratio † Number in each subgroup as per table 1. *adjusted odds ratio: adjusted for gender, age, marital status, social support, education, employment, household income, smoking, rurality, ever diagnosis of specified condition, except when the variable itself is being examined. ^R^= Referent group for odds ratios, except when the variable itself is being examined. ^R^= Referent group for odds ratios
