# Supplementary material for: Patterns of symptoms possibly indicative of cancer and associated help-seeking behaviour in a large sample of United Kingdom residents—The USEFUL study
Source: PLoS One. 2020 Jan 24;15(1):e0228033. doi: 10.1371/journal.pone.0228033 (PMC6980617; doi:10.1371/journal.pone.0228033)
Supplement: S1 File — (DOCX) [file pone.0228033.s003.docx]

**S1 File. Questionnaire content**

The full questionnaire asked about:

- experience of each symptom in the last year (occurrence; contact with GP about the symptom; whether respondent had experienced the symptom(s) before the last year);
- experience of each symptom in the last month (occurrence; when first started; duration of symptom(s);
- whether the symptom was still present;
- severity, interference, worry and overall bother when the symptom(s) was/were at its/their worst;
- contact with GP for the symptom(s) in the last month;
- actions taken for the symptom in the last month;
- views about the symptom which bothered the respondent most (based on the Brief Illness Perception Questionnaire [29]);
- views about going to see the GP (based on questions from the Cancer Awareness Measure [16]);
- socio-demographic characteristics;
- whether ever been diagnosed with asthma, cancer, epilepsy, chronic bronchitis/COPD, other chest disorder, heart disorder, stroke, diabetes, high blood pressure, liver disorder, arthritis/rheumatic disorder, mental health disorder, thyroid disorder, stomach/digestive disorder, other condition (to be specified);
- self-reported health;
- social support of the respondent.

A final section asked for permission to review the respondent’s medical records, and sought willingness to participate in a telephone interview and future studies. On-line and paper versions of the questionnaire were produced.
